# Effects of attention on the asymmetric serial dependences between form and motion patterns and their computational processes

**DOI:** 10.3389/fpsyg.2025.1505031

**Published:** 2025-04-09

**Authors:** Qian Sun, Si-Yu Wang, Meng-Ying Sun, Fan-Huan You, Ping Ran, Qi Sun

**Affiliations:** ^1^Department of Psychology, Zhejiang Normal University, Jinhua, China; ^2^Intelligent Laboratory of Zhejiang Province in Mental Health and Crisis Intervention for Children and Adolescents, Jinhua, China

**Keywords:** heading perception, form perception, attention, serial dependence, optic flow, Bayesian inference, information decay

## Abstract

Recent studies have revealed that serial dependences are asymmetric in the estimation of the focus of expansion (FoE) in the global static form and dynamic optic flow displays. In the current study, we conducted two experiments to examine whether and how attention affected the serial dependences between the two displays. The results showed that when all attentional resources are allocated to the FoE estimation task, the serial dependence of the form FoE estimation on the previous flow FoE (*SDE*_*flow*−*form*_) still existed even as the flow FoE was 40°, while the serial dependence of the flow FoE estimation on the previous form FoE (*SDE*_*form*−*flow*_) disappeared as the form FoE was beyond 30°. When attentional resources are distributed by other tasks, the *SDE*_*flow*−*form*_ tended to be stronger than the *SDE*_*form*−*flow*_. Therefore, the *SDE*_*flow*−*form*_ and *SDE*_*form*−*flow*_ are asymmetric regardless of observers' attentional states. Finally, we developed two Bayesian models to address the computational mechanism underlying the attentional effects. Both models proposed that attention modulated the certainty of sensory representations of currently presented features. In addition, the effects of working memory on previously presented features were considered in one model. The results showed that the Bayesian inference model that included working memory predicted participants' performances better than the model without considering working memory. In summary, the current study demonstrated that attention and working memory affected the serial dependences between form and flow displays, and the effects could be quantitatively predicted by Bayesian inference models.

## Introduction

The integration between dynamic and static visual features has always been a hot issue that attracts much research attention. At the beginning, researchers were led to think that the two types of features were processed independently and proposed a dual-pathway (“what” and “where”) model of information processing (DeYoe and Van Essen, 1988; Goodale and Milner, 1992; Milner and Goodale, 2006; Mishkin et al., 1983; Ungerleider and Mishkin, 1982). However, as research gets deep, more and more researchers now have come to understand that the processing of one feature encounters interference by another feature. That is, the two types of visual features are integrated with each other (Niehorster et al., 2010; Or et al., 2010; Pavan et al., 2017a; Wang et al., 2022, 2024; You et al., 2023), which has been supported by many neurophysiological and brain imaging studies (Kuai et al., 2020; Matsuyoshi et al., 2007; Pavan et al., 2017b; Tang et al., 2015).

**Figure 1:**
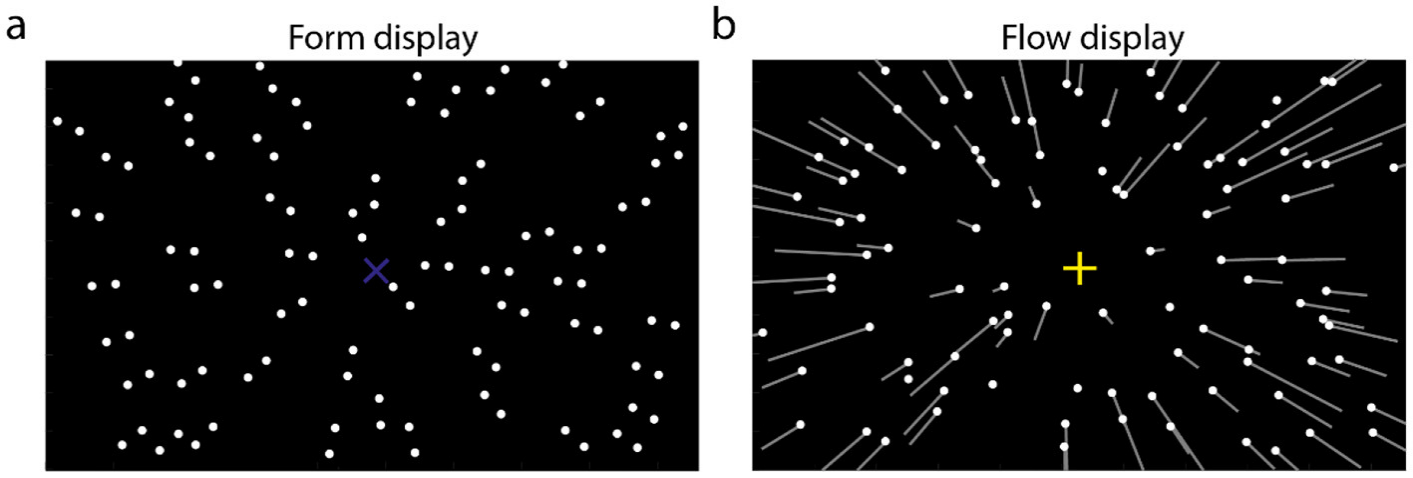
Stimulus displays used in the current study. **(a)** Form display consisted of dot pairs that are oriented to one position of the display (form FoE), illustrated by the blue “x.” **(b)** The flow display simulated observers translating in a 3D dot-cloud. White dots indicate the dots' position on the display in the first frame; white lines indicate the motion trajectories in the following frame. Yellow “+” indicates the heading direction (flow FoE). The white line, blue “x”, and yellow “+” are invisible in the experiments.

Previous studies have typically examined the integration of two types of simultaneously presented features (Kuai et al., 2020; Matsuyoshi et al., 2007; Niehorster et al., 2010; Or et al., 2010; Pavan et al., 2017a,b; Tang et al., 2015). However, given the fact that the natural world is stable and continuous in a short-time window, some researchers show their interests on information integration across the temporal domain. One of them is serial dependence, which was first proposed by Fischer and Whitney (2014). They found that the estimate of the currently presented orientation was biased toward the previously seen orientation (also see Kiyonaga et al., 2017; Manassi et al., 2023; Pascucci et al., 2023 for reviews). Inspired by their studies, Wang et al. (2022) alternatively showed participants form and flow displays in some conditions. The form display (Figure 1a) consisted of dot-pairs that converged at a position on the screen, forming a focus of expansion (i.e., form FoE). The flow displays (Figure 1b) simulated observers moving forward in a 3D dot-cloud, generating a dot-motion pattern that also contains a FoE (i.e., FoE). They found that the estimate of flow FoE was biased toward the previous form FoE, suggesting that the form and motion features can be integrated across the temporal domain.

Additionally, previous studies pointed out that the integration of simultaneously presented two features is asymmetric. That is, the effect of the form feature on the perception of motion feature was not equal to the effect of the flow feature on the perception of form feature (Or et al., 2010; Pavan et al., 2017a). You et al. (2023) alternatively presented form and flow displays and found the asymmetric serial dependences between form and flow displays, suggesting that the across-temporal integration between the two features is also asymmetric.

In addition to examining the integration of two features across the temporal domain, researchers also investigate the cognitive mechanisms involved in this integration. In the baseline condition of Wang et al. (2022), participants were shown a flow display and asked to estimate the flow FoE; in the perceptual condition, a form display was presented before the flow display and participants were asked to view the form display; in the memory-load condition, participants were asked to remember the form FoE and estimate it after estimating the flow FoE. The result showed the serial dependence sizes between the perceptual and memory-load conditions were not significantly different. This suggests that the serial dependence of the flow FoE estimation on the previous form display occurs at the sensory level and is information-driven. In a recent study, Wang et al. (2024) asked the participants to complete a number-addition task before estimating the form or flow FoE. The additional number-addition task distracted participants' attention, reducing the attentional resources allocated to estimating the form or flow FoE. They found that attention affected the serial dependence. Specifically, when the attentional load was on the flow FoE estimation, participants were more biased toward the previous form FoE estimate. In contrast, when the attentional load was on the form FoE estimation, the bias was reduced. Therefore, serial dependence is both information-driven and cognitive. In other words, the serial dependence occurs at both perceptual and post-perceptual levels.

Moreover, previous studies argue that serial dependence can be a Bayesian inference process. This means that when the reliability of current features is reduced, observers will bias their estimates more toward previously seen features, and vice versa (Cicchini et al., 2018; van Bergen and Jehee, 2019; Xu et al., 2022). Neurophysiological studies have shown that the neural activities of sensory cortices are affected by attention. When the attentional load is increased, these activities of the neurons to the stimuli are reduced and their tuning functions become less sensitive (Dubin and Duffy, 2007, 2009). As a result, the stimuli's representations are less reliable. Accordingly, it is expected that when the attentional load is on the current feature, the serial dependence on the previous feature will increase. Conversely, when the attentional load is on the previous feature, the serial dependence on the previous feature will decrease. These patterns have been found in several studies (Wang et al., 2022, 2024), indicating a Bayesian inference process. However, no study has developed a Bayesian inference model to directly examine this proposal.

In summary, the current study, designed based on Wang et al. (2024), conducted two experiments to examine the effect of attention on the asymmetric serial dependences between form and flow FoE estimations. Additionally, a Bayesian inference model was developed to directly examine the computational mechanism underlying the asymmetric serial dependences. The study reveals the manifestation of the asymmetric serial dependences and provides a basis for future neural computational models.

## Experiments: attention affects asymmetric serial dependence

### Methods

#### Participants

Forty participants were enrolled in our university. All participants had normal or corrected-to-normal vision and were naive to the purpose of the experiment. The study was in accordance with the declaration of Helsinki and was approved by the Scientific and Ethical Review Committee of our University. We obtained all participants' written informed consent form before starting the experiment. The participants were divided into two groups (Group 1: 8 men, 12 women; 18–25 years; Group 2: 8 men, 12 women; 18–24 years) and conducted Experiments 1 and 2, respectively. The sample size was decided based on previous studies (e.g., Wang et al., 2022, 2024; Xu et al., 2022).

#### Stimuli and apparatus

The current study consisted of two experiments: Experiments 1 and 2. In each experiment, participants were shown with two types of stimulus displays (112° H × 80° V; luminance of background: 0.24 cd/cm^2^). (1) Optic flow displays (Figure 1b) simulated observers moving forward along different directions in a 3D dot-cloud at a speed of 1 m/s. The 3D dot-cloud consisted of 90 dots (depth range: 0.2–10 m; diameter: 0.24°; luminance: 22.5 cd/cm^2^). From the observer's viewpoint, all dots appeared to radiate from a single point on the display, corresponding to the focus of expansion (FoE). The FoE was spatially aligned with the observer's self-motion direction in the 3D dot-cloud space, that is, heading direction. In Experiment 1, the flow FoE was randomly selected from the range of [−20°, 20°] with a step of 10°. Positive (negative) values indicate that flow FoEs are to the right (left) of the display center (0°). (2) Form displays (Figure 1a) consisted of 45 dot pairs that converged at a single position on the screen, referred to as the form FoE. In Experiment 1, the form FoE was positioned to the left or right of the flow FoE by 0°, 10°, or 20°, resulting in a range of form FoE positions from −40° to 40° with a step of 10°. Positive (negative) values indicate that form FoEs are to the right (left) of the display center (0°).

Additionally, in Experiment 2, the form FoE was selected from the range of [−20°, 20°] with a step of 10°. The flow FoE was positioned to the left or right of the form FoE by 0°, 10°, and 20°. As a result, the flow FoE was selected from the range of [−40°, 40°] with a step of 10°. Moreover, the difference between the form and flow FoEs was referred to as the FoE offset, which ranged from −20° to 20° in a step of 10°. In Experiment 1, positive (negative) values indicate that form FoEs are to the right (left) of flow FoEs. In Experiment 2, positive (negative) values indicate that flow FoEs are to the right (left) of form FoEs.

**Figure 2:**
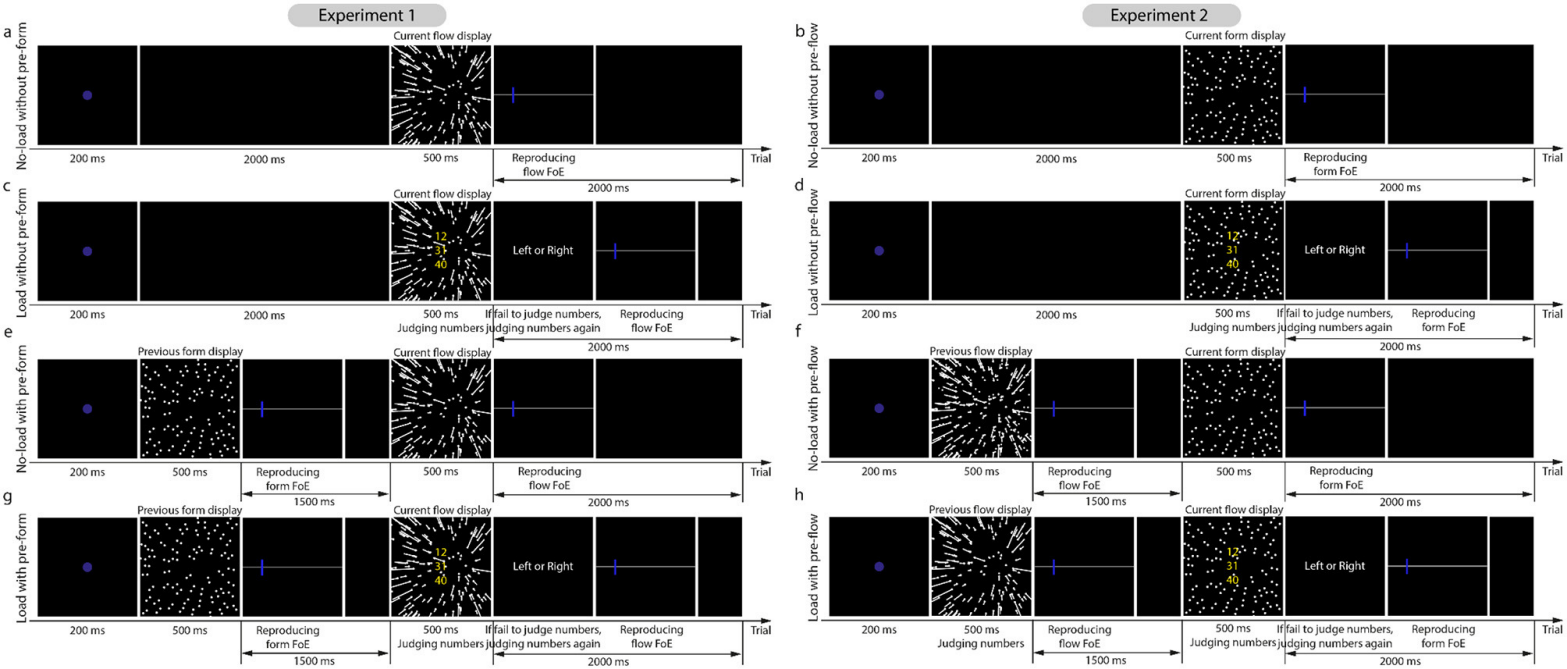
Illustrations of trial procedures in Experiments 1 and 2. **(a, b, e, f)** Illustrate the trail procedures in the non-load conditions. **(c, d, g, h)** Illustrate the trial procedures in the load conditions, in which, three integers were positioned vertically on the display center. Participants were asked to sum the first two integers up and compare the sum to the third integer.

Each experiment consisted of four blocks, with each corresponding to one condition (Figure 2): no-load without pre-display, load without pre-display, no-load with pre-display, and load with pre-display. In the two load conditions, three integers (RGB: [0, 0, 200]; 1.76° V × 1.76° H) were presented vertically at the center of the form or optic flow displays. The gap between the two numbers was 0.44°. Two random integers were chosen from the range of [11, 40] and the third integer was randomly chosen from the range of [40, 92]. Participants were asked to add the first two integers and compare the sum with the last integer. This resulted in a smaller allocation of attentional resources to the FoE estimation task in the load conditions compared to the no-load conditions.

The experiment was programmed using MATLAB with the Psychophysics Toolbox 3. Stimuli were displayed on a 27-inch ASUS monitor (resolution: 2,560 H × 1,440 V pixels; refresh rate: 60 Hz) with an NVIDIA GeForce GTX 1660Ti graphics card.

#### Procedure

The experiment was conducted in a dark room. The distance between the participants and the display center was 20 cm. Participants viewed the display monocularly with their right eyes to reduce the conflict between the motion parallax and binocular disparity depth cues. Participants' heads were stabilized using a chinrest, and they were instructed to maintain stillness in their eyes, head, and body to minimize the influence of non-visual information on heading estimation (Sun et al., 2020, 2022; Warren et al., 1988; Warren and Saunders, 1995; Xu et al., 2022).

As mentioned in stimuli and apparatus, each experiment consisted of four conditions: no-load without pre-display, load without pre-display, no-load with pre-display, and load with pre-display. As shown in Figure 2, the trial procedures of the two experiments were similar, except that Experiment 1 used the form display as the pre-display and the flow display as the current display, while Experiment 2 used the flow display as the pre-display and the form display as the current display.

Each trial of the load with pre-form condition in Experiment 1 (Figure 2g) started with a 200-ms fixation, followed by a 500-ms form display. Participants were then asked to report the position of the form FoE by moving a mouse-controlled probe on a horizontal line within 1,500 ms. If their response time was <1,500 ms, a blank display would be presented to make up the time. Following this, a 500-ms optic flow display was presented. Meanwhile, three integers were positioned at the center of the flow display. Participants were asked to add the first two integers up and compare the sum to the third integer as quickly and accurately as possible. If participants did not respond within 500 ms, a number reminder display was presented to remind them to complete the number-addition task. After responding, the participants reported the flow FoE by moving the mouse-controlled probe on the horizontal line. Note that the participants were asked to finish the number-addition task and the flow FoE estimation task within 2,000 ms. If they finished within 2,000 ms, a blank display was presented until the full duration of 2000 ms had elapsed. Then, the next trial began.

**Figure 3:**
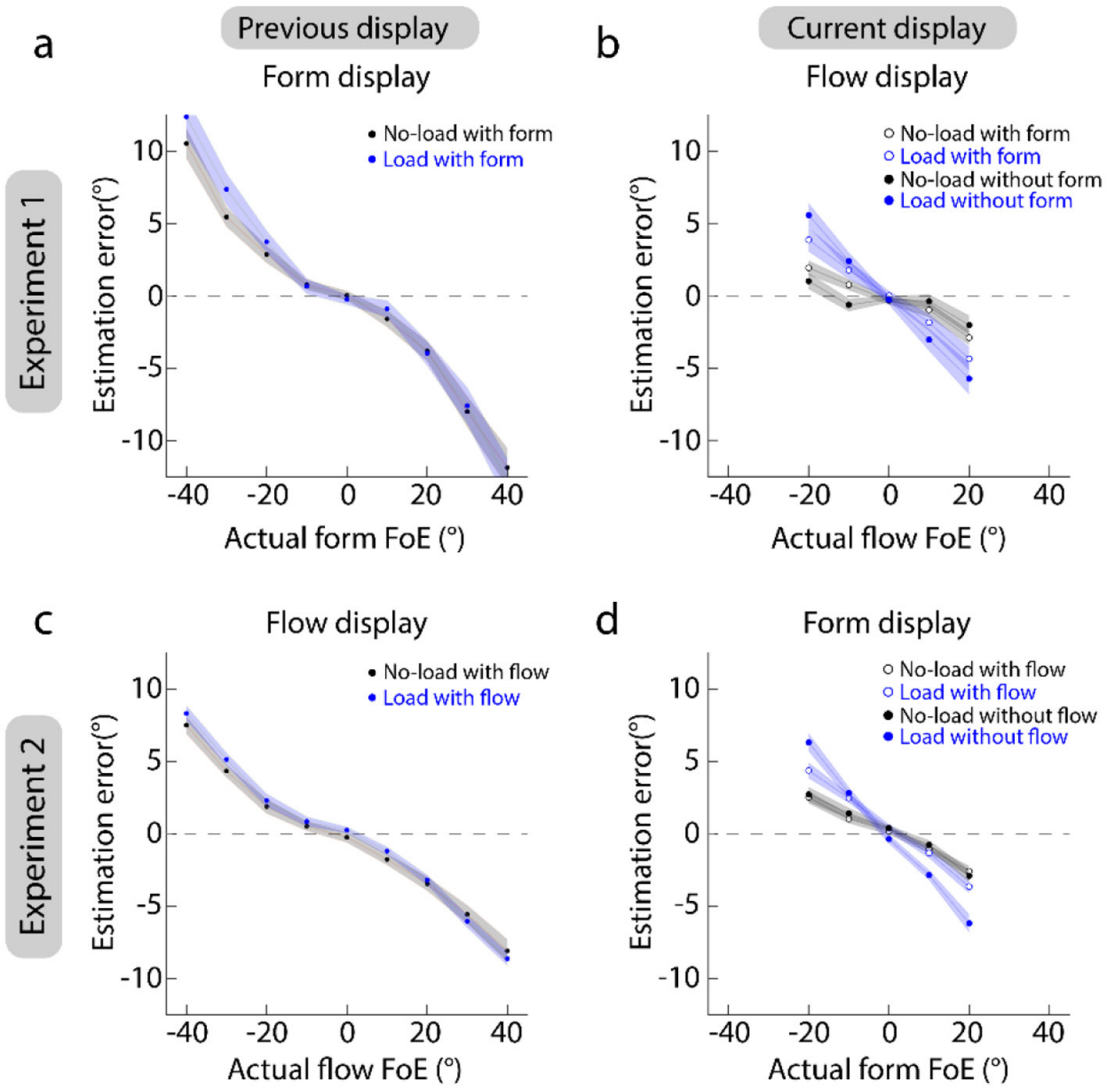
Results of estimation errors. **(a, b)** Show the results of Experiment 1. **(c, d)** Show the results of Experiment 2. Left panels show the estimation of previous displays. Right panels show the estimation of current displays. Dots and shaded areas indicate the mean and standard error of heading errors across 20 participants.

The trial procedures of other conditions (Figures 2a, c, and e) were developed on the above conditions. Specifically, (1) replacing the pre-form display and the form FoE response display with blank displays generated the load without pre-form condition (Figure 2c); (2) removing the number-addition task generated the no-load with pre-form condition (Figure 2e); (3) removing the number-addition task and the pre-form display generated the trial procedure of the no-load without pre-form condition (Figure 2a).

The trial procedures of the four conditions in Experiment 2 (right graphs in Figure 2) were similar to those in Experiment 1 (left graphs in Figure 2), except that the pre-display was the flow display and the current display was the form display.

The no-load and load without pre-display conditions included five current FoEs (0°, ±10°, and ±20°). Each current FoE was repeated 50 times. Thus, there were 250 trials (five current FoEs × 50 trials) in each condition. In no-load and load with pre-display conditions, each current FoE was accompanied by five FoE offsets (0°, ±10°, and ±20°)—the difference in the FoE between the previous and current displays. Each FoE offset was repeated 10 times. Thus, there were a total of 250 trials (five current FoE × five FoE offsets × 10 trials). Note that each block ideally contained 250 trials. If participants did not respond to the number-addition task or the heading estimation task within the allotted time, the trial would be added back to the trial list. As a result, some participants completed more than 250 trials in some blocks.

Participants were given approximately 15 practice trials before each condition block to familiarize themselves with the condition. The corresponding block then started and lasted for about 20 min. The conducting sequences of the four conditions were counterbalanced across participants.

#### Data analysis

In the load (with or without pre-display) conditions of each experiment, we first calculated the accuracy of the number-addition task. Participants with an accuracy below 0.75 were removed. The results showed that all participants had an accuracy above 0.75. Trials where participants did not complete the number-addition or heading-estimation tasks within the specified time interval were excluded. As a result, each participant had 250 trials in each block.

For the form or flow FoE estimation tasks, we recorded the participants' FoE estimates and calculated the difference between the estimated and actual FoEs, which we named estimation error (EE). The estimation accuracy decreases as the absolute value of EE increases. To examine whether the attentional load affected the FoE estimation, a 2 (load conditions: load vs. no-load) × 9 (actual FoEs: 0°, ±10°, ±20°, ±30°, and ±40°) repeated measures ANOVA was conducted for the pre-form and pre-flow displays, and a 2 (load conditions: load vs. no-load) × 5 (actual FoEs: 0°, ±10°, and ±20°) × 2 (pre-displays: with vs. without) repeated measures ANOVA was conducted for the current form and flow displays.

To examine whether the current flow FoE estimation relied on the previously presented form display, we first calculated the difference in the estimated FoE between the previous and current displays in the no-load with pre-display conditions. This difference was named the FoE offset, with values of 0°, ±10°, and ±20°. Therefore, each current FoE (0°, ±10°, and ±20°) was paired with five FoE offsets, resulting in 25 combinations. Then, the estimation error of each current FoE in each FoE offset was calculated (*EE*_*no*−*load*−*with*−*pre*−*form*_) and then subtracted from the estimation error of the corresponding current FoE in the no-load without pre-form conditions (*EE*_*no*−*load*−*without*−*pre*−*form*_). This resulted in the residual estimation error in the no-load condition (*REE*_*no*−*load*_). It was given by:


(1) $$\begin{array}{l} REE_{no-load}=EE_{no-load_{-}with_{-}pre-form}\\ \;-EE_{no-load_{-}without_{-}pre-form}, \end{array}$$

Similar procedures were adopted to calculate the residual estimation error in the load condition (*REE*_*load*_). It was given by:


(2) $$\begin{array}{l} REE_{load}=EE_{load_{-}with_{-}pre-form}\\ \;-EE_{load_{-}without_{-}pre-form}, \end{array}$$

If the *REE*_*no*−*load*_ or *REE*_*load*_ was significantly different from 0, then the current flow FoE estimation was affected by the previous form FoE. Additionally, if the *REE*_*no*−*load*_ or *REE*_*load*_ shared the same sign with the FoE offset, then the current flow FoE estimate was biased toward the previous form FoE, indicating an attractive seral dependence.

To examine whether attention affected the serial dependence of the flow FoE estimation on the previous form FoE, a 2 (load conditions: load vs. no-load) × 5 (actual FoEs: 0°, ±10°, and ±20°) × 5 (FoE offsets: 0°, ±10°, and ±20°) repeated measures ANOVA was conducted.

Next, similar procedures were adopted to examine the serial dependence of the form FoE estimation on the previous flow FoE in Experiment 2.

### Behavioral results

#### Estimation accuracy

Figures 3a, c plot the estimation error against the actual FoE of pre-form and pre-flow displays. They show that the estimation errors in the no-load and load conditions are overlapped. A 2 (load conditions: load vs. no-load) × 9 (actual FoEs: 0°, ±10°, ±20°, ±30°, and ±40°) repeated measures ANOVA showed that the absolute estimation errors were not significantly different between the load and no-load conditions [Experiment 1: *F*_(1, 19)_ = 3.44, *p* = 0.079, partial η^2^ = 0.15; Experiment 2: *F*_(1, 19)_ = 1.95, *p* = 0.18, partial η^2^ = 0.093]. This suggests that the attentional load presented after the stimulus displays does not affect the estimation of form and flow FoEs.

Figures 3b, d plot the estimation error against the actual FoE of current-form and current-flow displays. They show that the absolute estimation errors in the load conditions (blue markers) are higher than those in the no-load conditions (black markers). A 2 (load conditions: load vs. no-load) × 5 (actual FoEs: 0°, ±10°, and ±20°) × 2 (pre-displays: with vs. without) repeated measures ANOVA showed that the interaction between actual FoEs and load conditions was significant [Greenhouse-Geisser corrected: Experiment 1: *F*_(1.21, 22.99)_= 17.42, *p* < 0.001, partial η^2^= 0.48; Experiment 2: *F*_(1.56, 26.70)_ = 43.68, *p* < 0.001, partial η^2^= 0.70]. Furthermore, simple-effect test with Bonferroni correction showed that when the FoE deviated from the display center (0°), the absolute estimation errors in the load condition were significantly higher than those in the no-load conditions for (*p*s < 0.0088). Additionally, in Experiment 2, the interaction between pre-displays and load conditions was also significant [Greenhouse-Geisser corrected: *F*_(1.47, 27.91)_ = 8.07, *p* = 0.0038, partial η^2^= 0.30]. Furthermore, a simple-effect test with Bonferroni correction showed that only when the pre-flow display was presented, the absolute estimation error in the no-load condition (Mean ± SE: 0.071 ± 0.36) was marginally smaller than that in the load condition (0.93 ± 0.53) (*p* = 0.062). Together, these results suggest that the estimation of form and flow FoEs was affected by the attentional load. When the attentional resources were distracted by irrelevant tasks (e.g., the number-addition task), the FoE estimation accuracy was reduced.

Additionally, comparing Figures 3a, c shows that the absolute estimation errors for form FoE estimation are generally larger than those for flow FoE estimation. A repeated measures ANOVA with load conditions and actual FoEs as the within-subject factor and experiments as the between-subject factor showed that the interaction between actual FoEs and experiments was significant [Greenhouse-Geisser corrected: Experiment 1: *F*_(1.32, 50.03)_ = 6.44, *p* = 0.0088, partial η^2^ = 0.15]. Furthermore, a simple-effect test with Bonferroni correction showed that when the FoEs were ±30° and ±40°, the absolute estimation errors for form FoE estimation tended to be significantly larger than those for flow FoE estimation (*p*s < 0.010), suggesting that the estimation of form FoEs was harder than that of flow FoEs when the FoE was beyond the 30°.

#### Serial dependence

**Figure 4:**
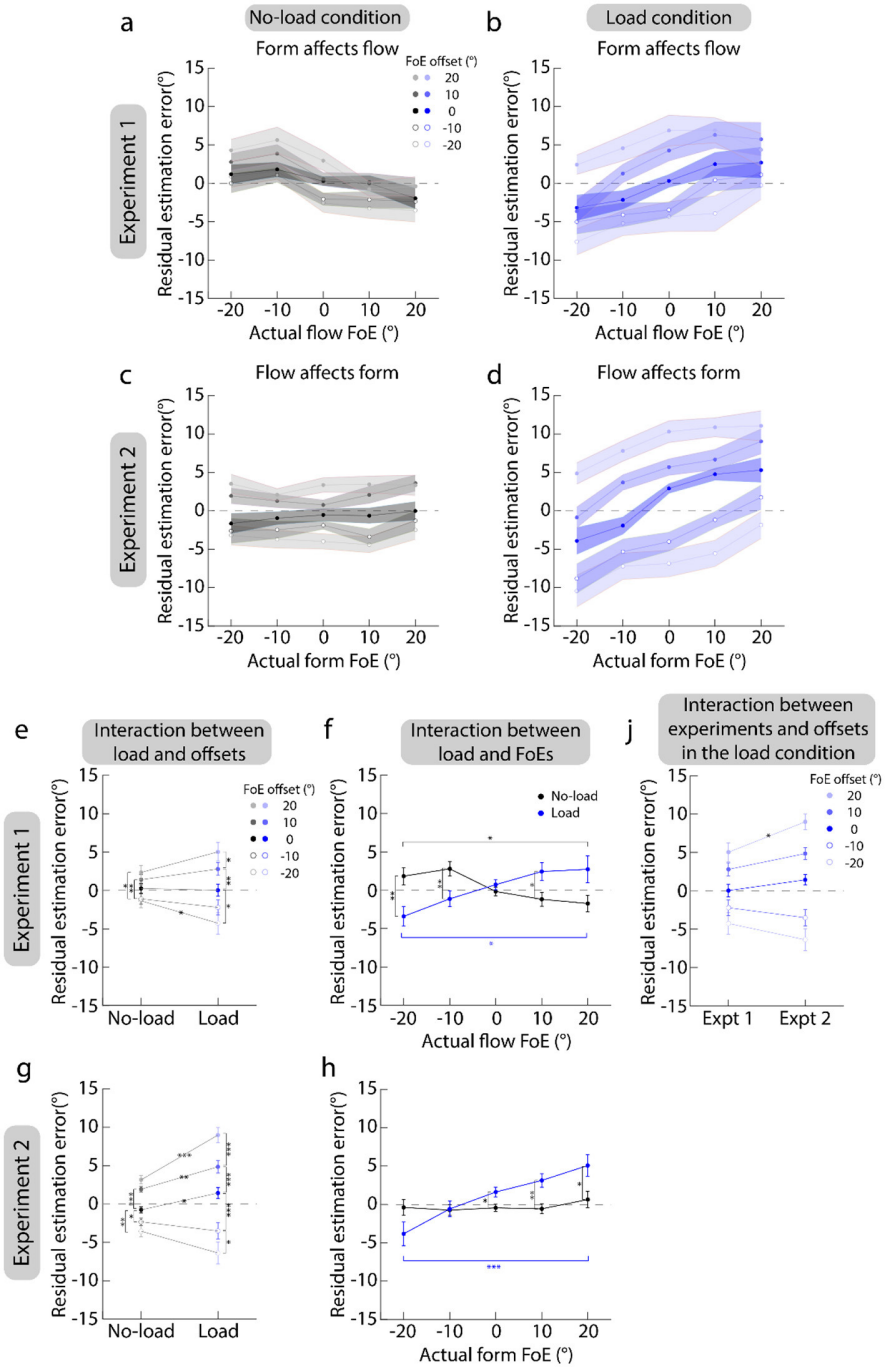
Results of serial dependence. **(a–d)** Plot the residual estimation error against the actual FoEs. **(a, b)** Show the results of Experiment 1. **(c, d)** Show the results of Experiment 2. Left panels show the serial dependence in the no-load condition. Right panels show the serial dependence in the load condition. Dots and shaded areas indicate the mean and standard error of heading errors across 20 participants. **(e, g)** Show the interaction between load conditions (no-load vs. load) and FoE offsets. **(f, h)** Show the interaction between load conditions (no-load vs. load) and actual FoEs. **(j)** Shows the interaction between experiments (Experiments 1 vs. 2) and FoE offsets in the load condition. In **(e–j)**, error bars are the standard errors across 20 participants. ^*^p < 0.05; ^**^p < 0.01; ^***^p < 0.001.

Figures 4a–d plot the residual estimation error against the actual flow FoE. They show that the residual estimation error overall shares the same sign with the actual FoE (also see Figures 4e, g). That is, the residual estimation error is negative as the FoE offset is negative (circle markers) and vice versa (light solid dots). This suggests that the flow (form) FoE estimates are biased toward the previously seen form (flow) display in Experiment 1 (2). A 2 (load conditions: load vs. no-load) × 5 (actual FoEs: 0°, ±10°, and ±20°) × 5 (FoE offsets: 0°, ±10°, and ±20°) repeated measures ANOVA showed that the main effects of FoE offsets were all significant [Greenhouse-Geisser corrected: Experiment 1, *F*_(1.23, 23.43)_ = 20.55, *p* < 0.001, partial η^2^ = 0.52; Experiment 2, *F*_(1.22, 23.09)_ = 75.48, *p* < 0.001, partial η^2^ = 0.80]. Furthermore, the *post-hoc* analysis with Bonferroni correction showed that in Experiment 1 (Figure 4e), the estimation errors of ±10° and ±20° FoE offsets were significantly larger than that of 0° (*p*s < 0.011), and the estimation error of the 20°-FoE offset was also significantly larger than that of the 10°-FoE offset (*p* = 0.026); in Experiment 2 (Figure 4g), the estimation errors of ±10° and ±20° FoE offsets were significantly larger than that of 0° (*p*s < 0.001), and the estimation errors of ±20° FoE offset was also significantly larger than that of ±10° FoE offset (*p*s < 0.0026). These results indicate that attractive serial dependences are between form and flow FoE estimations.

Additionally, the interactions between load conditions and FoE offsets were significant [Greenhouse-Geisser corrected: Experiment 1, *F*_(1.23, 23.43)_ = 20.55, *p* < 0.001, partial η^2^ = 0.52; Experiment 2, *F*_(1.22, 23.09)_ = 75.48, *p* < 0.001, partial η^2^ = 0.80]. Furthermore, a simple-effect test with Bonferroni correction showed that the residual estimation error tended to be larger in the load condition than in the no-load condition (Figures 4e, g). This suggests that when the attention resources allocated to the estimation task were reduced, the observers relied more on the previously seen display, showing a stronger serial dependence.

Moreover, the interactions between load conditions and actual FoEs were also significant [Greenhouse-Geisser corrected: Experiment 1, *F*_(1.64, 31.16)_ = 9.05, *p* = 0.0015, partial η^2^ = 0.32; Experiment 2, *F*_(1.57, 29.74)_ = 6.38, *p* = 0.0082, partial η^2^ = 0.25]. The simple-effect test with Bonferroni correction showed that in the no-load condition of Experiment 1 (black markers in Figure 4f), the residual estimation errors were positive when the actual flow FoEs were negative, and vice versa. This suggested that form FoEs close to the display center had a stronger effect on flow FoE estimation than those farther away. In the load condition (blue markers in Figure 4f), the opposite trend was observed, indicating that the form FoEs, which are further away from the display center, had a stronger effect on the estimation of flow FoE than those that are closer to the display center.

In the no-load condition of Experiment 2 (black markers in Figure 4h), the residual estimation errors were not significantly different among different form FoEs (*x*-axis), showing that the sizes of serial dependences were very close for the flow FoEs that were symmetric about the form FoE. For example, when the form FoE was −20°, the absolute values of residual estimation errors were 3.18° (SE: 1.27°, 95% CI”: [0.53°, 5.84°] and 3.53° (SE: 1.23°, 95% CI: [0.96°, 6.10°]) for the −40° and 0° flow FoEs. Their difference was not significant. The trend in the load condition (blue markers in Figure 4h) was consistent with that in Experiment 1, indicating that the flow FoEs further away from the display center had a stronger effect on the estimation of form FoE than those closer to the display center.

Next, comparing Figures 4b, d shows that the residual estimation errors tend to be larger in Experiment 2 than in Experiment 1. To test this trend, a three-factor repeated measures ANOVA with experiments (1 vs. 2) as a between-subject factor was conducted. The results showed that the interaction between experiments and FoE offsets was significant [Greenhouse-Geisser corrected: *F*_(1.24, 47.22)_ = 4.47, *p* = 0.032, partial η^2^ = 0.11]. Furthermore, simple-effect test with Bonferroni correction showed that only when the FoE offset was 20°, the residual estimation error of Experiment 2 was significantly larger than that of Experiment 1 (*p* = 0.017).

In summary, the results suggest that attractive serial dependencies are between the form and flow FoE estimations, which are affected by attention. Furthermore, when comparing the serial dependence of the flow FoE estimation on the previous form FoE (*SDE*_*form*−*flow*_, Experiment 1), the serial dependence of the form FoE estimation on the previous flow FoE (*SDE*_*flow*−*form*_, Experiment 2) is stronger. Specifically, when all attentional resources are allocated to the FoE estimation task, the *SDE*_*flow*−*form*_ exists even as the flow FoE is 40°, while the *SDE*_*form*−*flow*_ disappears as the form FoE is beyond 30°. When attentional resources are distracted by other tasks, the *SDE*_*flow*−*form*_ tends to be higher than the *SDE*_*form*−*flow*_. Therefore, the *SDE*_*flow*−*form*_ and *SDE*_*form*−*flow*_ are asymmetric.

### Bayesian inference models

Previous studies have revealed that serial dependence is consistent with a Bayesian inference process (Cicchini et al., 2018; van Bergen and Jehee, 2019; Xu et al., 2022). This means that observers depend more on the previously seen feature to estimate the current feature as the reliability of the current feature decreases or reduce the dependence on the previous feature as the reliability of the current feature increases. In addition, previous studies have revealed that the form FoE can affect the estimation of the flow FoE, and vice versa (Niehorster et al., 2010; You et al., 2023). Previous neurophysiological studies have also revealed that the cortical areas V3a and V3b/KO could be involved in the integration of the two FoEs (Kuai et al., 2020). Therefore, it is reasonable to take the previously presented form feature as the prior of the flow FoE.

The analysis above showed that the attentional load reduced the accuracy of current FoE estimation and biased the estimation of current FoEs more toward previous feature, which well matches the Bayesian inference process. Next, we developed a Bayesian inference model to quantitatively examine the proposal.

### Methods

Our model consisted of two layers. Layer 1 predicted FoE estimates of form and flow displays in the load and no-load conditions. This layer generated the likelihoods of different FoEs without serial dependence. Layer 2 predicted the residual estimation error induced by serial dependence. The details of each layer are provided below.

#### Layer 1: generating likelihood distributions

According to the Bayesian inference theory, the final estimate (*m*) of one feature (θ) is from a posterior distribution (*p*(θ|*m*)) that is the optimal combination of the prior distribution (*p*(θ)) and likelihood distribution (*p*(*m*|θ)). It can be given by:


(3) $$\begin{array}{l} p\left(\theta |m\right)\propto p\left(\theta \right)\cdot p\left(m|\theta \;\right). \end{array}$$

In the current study, the participants were shown with the form and flow displays. We hypothesized that the prior distributions (*p*(θ)) about form and flow FoEs were the same and could be depicted by a Gaussian function:


(4) $$\begin{array}{l} p\left(\theta \right)\;=\frac{1}{\sqrt{2\pi }\sigma_{p}}e^{\left(\frac{-{\left(\theta -\;0\right)}^{2}}{2{\sigma_{p}}^{2}}\right)}, \end{array}$$

where σ_*p*_ indicates the standard deviation of the prior. For computational convenience, it was given by tuning curve of the population activities of MSTd neurons (Chen et al., 2008). That is, σ_*p*_ was a constant.

Additionally, the likelihood distributions (*p*(*m*|θ)) were also given by a Gaussian function, given by:


(5) $$\begin{array}{l} p\left(m|\theta \right)=\frac{1}{\sqrt{2\pi }\sigma_{l}}e^{\left(\frac{-{\left(m-\theta \right)}^{2}}{2{\sigma_{l}}^{2}}\right)}, \end{array}$$

where σ_*l*_ represents the standard deviation of the likelihood distribution. Its value varied systematically across the experimental conditions, such as (1) stimulus type (form vs. flow displays), (2) cognitive load (load vs. no-load conditions), and (3) focus of expansion (FoE) locations. Consequently, σ_*l*_ was modeled as a set of free parameters that were independently estimated for each unique combination of experimental conditions, stimulus types, and FoE positions.

The final estimate ($\hat{m}$) of each FoE (θ) was given by:


(6) $$\begin{array}{l} \hat{m}\left(\theta \right)=\int_{-180}^{180}p\left(\theta |m\right)\cdot xdx, \end{array}$$

Therefore, Layer 1 consists of two types of parameters {σ_*p*_, σ_*l*_}, representing standard deviation of prior and likelihood distribution. Note that σ_*l*_ varies across different load conditions, stimulus types and FoEs. The best parameter values were decided by the least square error methods. That is


(7) $$\begin{array}{l} min\left({\left|\hat{m}-m\right|}^{2}\right), \end{array}$$

Moreover, the above procedures finally generated the predicted estimate error for the form and flow displays with different FoEs in the load and no-load conditions.

#### Layer 2: predicting residual estimation error induced by serial dependence

Layer 1 provides the likelihood distributions of different FoEs in the load and no-load conditions and the predicted estimation error without previous displays (${\hat{EE}}_{no-load_{-}without_{-}pre-display}$ and ${\hat{EE}}_{load_{-}without_{-}pre-display}$). Next, Layer 2 predicted the estimation error with previous displays (${\hat{EE}}_{no-load_{-}with_{-}pre-display}$ and ${\hat{EE}}_{load_{-}with_{-}pre-display}$).

Given that the serial dependence means the bias of the estimate of current features toward previous features, the prior distributions also included the likelihood distribution of previous display aside from the prior (θ). The posterior was given by:


(8) $$\begin{array}{l} p\left(\theta_{c}|m\right)\propto p\left(\theta_{c}\right)\cdot p\left(m|\theta_{c}\right)\cdot p\left(m|\theta_{p}\right), \end{array}$$

where subscripts *c* and *p* indicate the current and previous FoEs.

It was noted that a 1,500-ms time interval existed was between the previous and current displays. Hence, the FoE of the previous display should be firstly stored in the working memory and then integrated with the current display (Sun et al., 2023). While in the working memory, the representation of the previous FoE could be changed. Consequently, the standard deviation of the likelihood when the feature was currently presented differed from when the feature was previously presented. Therefore, we introduced a scaling factor (*a*) to represent the effects of working memory. That is, the standard deviation of previous display (σ_*l*_−_*p*_) was the product of *a* and the standard deviation of current display (σ_*l*_−_*c*_), given by:


(9) $$\begin{array}{l} \sigma_{l_{-}p}=a\cdot \sigma_{l_{-}c}, \end{array}$$

Replacing the posterior in Equation (6) with Equation (8) and repeating Equations (6) and (7), we would get the predicted estimation error with previous displays (${\hat{EE}}_{no-load_{-}with_{-}pre-display}$ and ${\hat{EE}}_{load_{-}with_{-}pre-display}$). We then use them to subtract the predicted estimation error without previous displays (${\hat{EE}}_{no-load_{-}without_{-}pre-display}$ and ${\hat{EE}}_{load_{-}without_{-}pre-display}$) to get the predicted residual estimation error (${\hat{REE}}_{no-load}$ and ${\hat{REE}}_{load}$).

Therefore, in Layer 2, there is a free parameter {*a*} indicating the working memory effect. To optimize the parameter estimation, we implemented a least-squares minimization approach, which minimizes the squared differences between the model-predicted residual estimation errors and the empirically observed residual estimation errors, given by


(10) $$\begin{array}{l} min\left({\left|\hat{REE}-REE\right|}^{2}\right), \end{array}$$

Aside from the above model, we also developed another model without considering the effects of working memory. That is, the standard deviation of previous display (σ_*l*_−_*p*_) was equal to the standard deviation of current display (σ_*l*_−_*c*_), given by:


(11) $$\begin{array}{l} \sigma_{l_{-}p}=\sigma_{l_{-}c}. \end{array}$$

If the model involving working memory predicted participants' data better than the model excluding working memory, then the working memory played a role in serial dependence.

### Results

**Figure 5:**
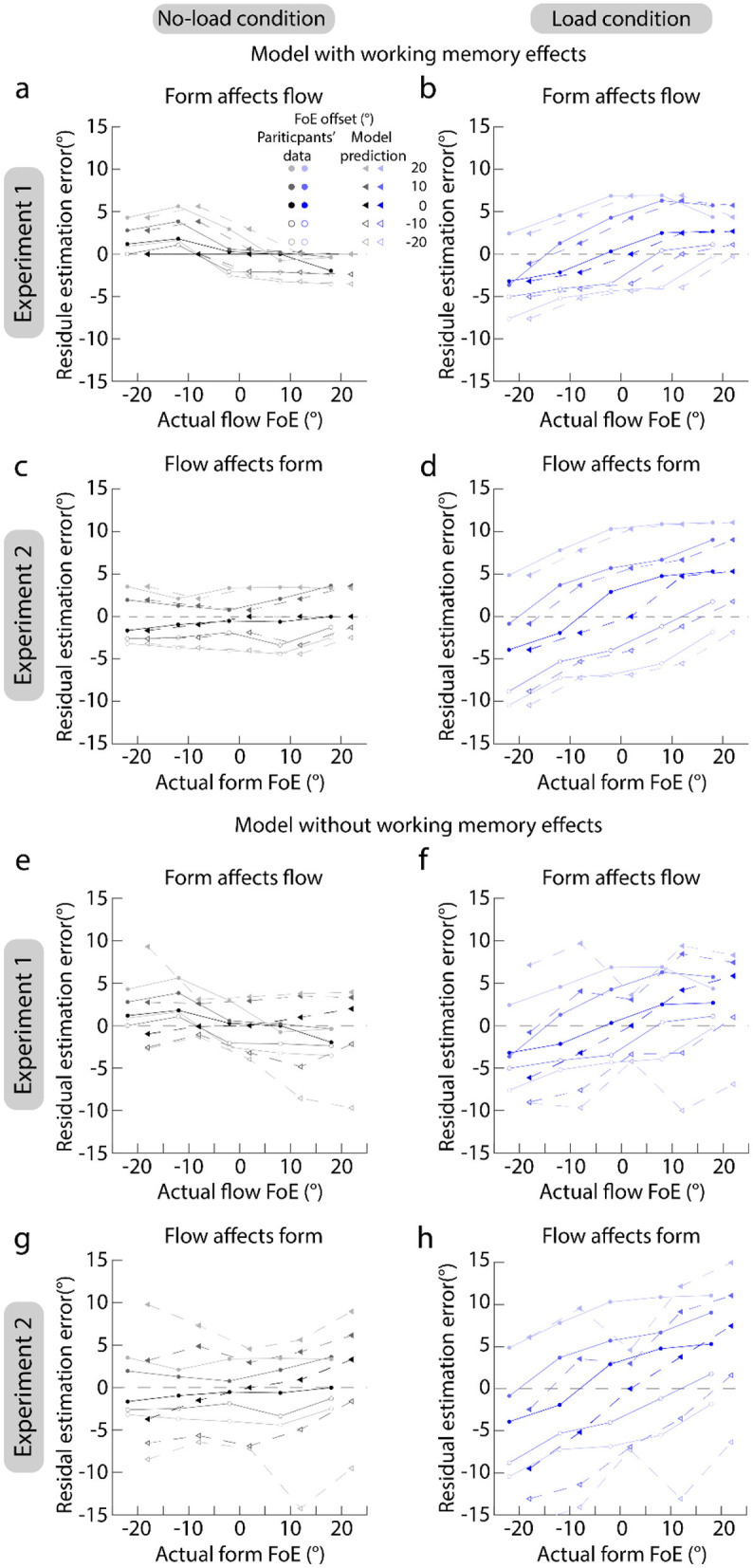
Results of serial dependence predicted by models. **(a–d)** Show the predicted results of model which considered the effect of working memory. **(e–h)** Show the predicted results of model which did not consider the effect of working memory. **(a, b, e, f)** Show the results of Experiment 1. **(c, d, g, h)** Show the results of Experiment 2. Left panels show the serial dependence in the no-load condition. Right panels show the serial dependence in the load condition. To clearly show the data of participants and model predictions, we shifted their actual FoEs to the left or right side by 2°.

Figure 5 plots the serial dependence results of participants (dots and circles) and model predictions (triangles). Figures 4a–d clearly show that the predictions of model involving the working memory well match participants' data. This suggests that the serial dependences between the form and flow FoE estimations are consistent with a Bayesian inference process even as the attentional resources allocated to the FoE estimation task were reduced, and the attentional load affects serial dependence by reducing the certainty of the feature representation.

However, compared with the predictions of the model involving working memory, the predictions of the model excluding working memory were deviated from the participants' data a lot (Figures 5e–h). This suggests that working memory is also involved in serial dependence.

## Discussion

In the current study, two experiments were conducted and it was found that the serial dependences between the flow and form FoE estimations were asymmetric. Specifically, when all attentional resources are allocated to the FoE estimation task, the serial dependence of the form FoE estimation on the previous flow FoE (*SDE*_*flow*−*form*_) still existed even as the flow FoE was 40°, while the serial dependence of the flow FoE estimation on the previous form FoE (*SDE*_*form*−*flow*_) disappeared as the form FoE was beyond 30°; however, when attentional resources are distracted by other tasks, the *SDE*_*flow*−*form*_ tended to be stronger than the *SDE*_*form*−*flow*_. These findings enrich the asymmetric serial dependences revealed by directly varying the displays (You et al., 2023). Additionally, the asymmetric serial dependences and the attentional effects were well predicted by a Bayesian inference model, revealing the underlying computational mechanisms.

Wang et al. (2024) is the first study to examine the effects of attention on the serial dependence of the flow FoE estimation on previous form display. Our current study extended their research and revealed the effects of attention on the serial dependence of form FoE estimation on previous flow display. Taken together, these findings, we point out that serial dependence between form and motion features are cognitive rather than solely information-driven (Wang et al., 2022). In addition, this finding further enriches the evidence for the proposal that serial dependence occurs at different stages of perception (Bae and Luck, 2020; Ceylan et al., 2021; Fritsche et al., 2017; Pascucci et al., 2019; Sun et al., 2023; Xu et al., 2022).

You et al. (2023) alternatively presented form and flow displays and varied the dot densities of two displays. They found that the pattern of serial dependence of the flow FoE estimation on the previous form, along with the change of stimulus reliabilities, differed from that of the serial dependence of the form FoE estimation on the previous flow, indicating asymmetric serial dependences. Previous studies have shown that serial dependence occurs within a 12-s time window, meaning that only the two stimuli presented within that time window can be integrated (Fischer and Whitney, 2014; Manassi et al., 2023). In You et al. (2023), there are at least three flow and three form displays presented in that time. As a result, the estimation of current flow was affected by both previous form and flow displays. Hence, their conclusions should be re-examined. Our current study used a block design and calculated the difference in the estimation error between with and without pre-display conditions (Figure 2). This ensures that the left estimation error is solely induced by previous displays or serial dependence. Therefore, we confirmed the asymmetric serial dependences between the two displays using a more rigorous experimental design.

Additionally, we identified the asymmetric patterns in different attentional states. Firstly, when all attentional resources were focused on the estimation task (Figures 4a, c), the current form FoE estimation was affected by the flow FoE even at 40°. In contrast, the current flow FoE estimation was only affected by the form FoE only when it was <30°. These findings suggest that the range of effects of flow display on form display is wider than the range of effects of form display on flow display. Previous studies have demonstrated some neurons in several cortices respond to form and motion stimuli simultaneously, such as V1 (Adelson and Movshon, 1982), KO (Kuai et al., 2020; Pavan et al., 2017b) and MT+ (Matsuyoshi et al., 2007; Tang et al., 2015), and the receptive fields of which have smaller receptive fields when responding to form stimuli (De Valois, 1977) compared to when responding to motion stimuli (Albright, 1984). Integration only occurs when the neurons with overlapping receptive fields capture both the form and flow FoEs. Therefore, it can be proposed that in our current study, the previous form display with the 40° FoE falls outside of the neurons' receptive field when the neurons respond to the form display with the 20° FoE. In contrast, the previous flow display with the 40° FoE falls inside of the neurons' receptive field when the neurons responding to the flow display with the 20° FoE.

Secondly, when the attentional resources allocated to the FoE estimation of current features were reduced (Figures 4b, d), the effect size of previous flow displays on the current form FoE estimation tends to be higher than that of previous form displays on the current flow FoE estimation. These suggest that the representation of flow displays is more reliable than that of form displays. As shown in Figure 3, the estimation accuracy of flow displays was higher than that of form displays, supporting our proposal.

Moreover, previous studies have demonstrated that working memory is involved in serial dependence (Bae and Luck, 2020; Bliss et al., 2017; Fritsche et al., 2017; Sun et al., 2023). Furthermore, the attentional load can reduce the representation precision of stimuli stored in working memory (Allen et al., 2017; Souza and Oberauer, 2017). In the current study, the numbers used to distract participants' attention were presented on the display center (Figure 2). As a result, the representation reliabilities of previous displays are reduced. According to the Bayesian inference theory, it is reasonable that the biases toward the FoEs of these previous displays are decreased (Figures 4b, d).

Another important contribution of the current study is that our Bayesian inference model well predicts the effects of the attentional load on serial dependence, suggesting that the attentional effect is consistent with a Bayesian inference process from the computational perspective. Previous studies only proposed that the serial dependence effect between form and flow information was consistent with the Bayesian inference process based on the trend of the data but did not examine it by developing a computational model (Wang et al., 2024; You et al., 2023). In addition, our model proposes that the attentional load modulates the standard deviation of likelihood distributions that are positively correlated with the width of sensory neurons' tuning curves (Ma et al., 2008; Stocker and Simoncelli, 2006; Butts and Goldman, 2006). Previous neurophysiological studies have found that the tuning widths increase with the increase in the attentional load (Dubin and Duffy, 2007, 2009). Our model suggested that the standard deviation of likelihood distributions in the load condition were larger than those in the no-load condition (Table 1).

**Table 1:** The standard deviations of likelihood distributions in the no-load and load conditions.

		**FoE(°)**				
		**−20**	**−10**	**0**	**10**	**20**
Flow display	No-load	6.18	3.12	2.39	7.15	8.55
	Load	15.05	12.90	2.71	16.72	15.57
Form display	No-load	9.05	6.56	2.55	7.02	9.95
	Load	17.66	15.35	2.56	15.37	17.07

Aside from the effects of attention, our models also took the effects of studying memory into account. Compared with the predictions of model without the studying memory parameter, the model with the parameter well predicted participants' performance (Figure 5). Hence, our model implies that studying memory and attention simultaneously affect serial dependence. Their roles were discussed independently in different studies (Bae and Luck, 2020; Bliss et al., 2017; Fischer and Whitney, 2014; Fritsche and de Lange, 2019; Fritsche et al., 2017; Sun et al., 2023; Wang et al., 2024). Additionally, we also admit that the above conclusion is made based on the computational modeling. From the perspective of modeling, a model with an extra parameter can overfit the data regardless of the parameters' psychological mechanisms, which makes sense. However, the proposal that the parameter represents studying memory is a direction for future studies.

It should be noted that compared with the non-load condition, the numbers in the load condition could guide participants' gaze direction. As a result, the gaze would be more such asly to be fixed on the display center in the load condition than in the no-load condition. In other words, there were more free gazes in the no-load condition than in the load condition. Moreover, the number-addition task could lead to the longer onset time for the estimation task than in the no-load condition, which could lead to more internal noise that cannot be totally attributed to attention. Hence, these two confounding factors (gaze and delay) could reduce the reliability of our conclusions about estimation standard deviation, which are good open questions for future studies.

In summary, the current study systematically addresses the effects of attention on the asymmetric serial dependences between static form and dynamic flow features and reveals the underlying computational mechanisms. These conclusions are made based on the behavioral and computational modeling evidence, some of which are only hypotheses. Future studies can employ neurophysiological and brain imaging techniques to further investigate these findings.

## Data Availability

The raw data supporting the conclusions of this article will be made available by the correspondence author (Qi Sun, sunqi_psy@zjnu.edu.cn), without undue reservation.

## References

[B1] Adelson E. H. Movshon J. A. (1982). Phenomenal coherence of moving visual patterns. Nature 300, 523–525. 10.1038/300523a0 7144903

[B2] Albright T. D. (1984). Direction and orientation selectivity of neurons in visual area MT of the macaque. J. Neurophysiol. 52, 1106–1130. 10.1152/jn.1984.52.6.1106 6520628

[B3] Allen R. J. Baddeley A. D. Hitch G. J. (2017). Executive and perceptual distraction in visual studying memory. J. Exp. Psychol. Human Percep. Perform. 43, 1677–1693. 10.1037/xhp0000413 28414499 PMC5560518

[B4] Bae G. Y. Luck S. J. (2020). Serial dependence in vision: merely encoding the previous-trial target is not enough. Psychonom. Bull. Rev. 27, 293–300. 10.3758/s13423-019-01678-7 31898266 PMC7101255

[B5] Bliss D. P. Sun J. J. D'Esposito M. (2017). Serial dependence is absent at the time of perception but increases in visual studying memory. Sci. Rep. 7, 1–13. 10.1038/s41598-017-15199-7 29116132 PMC5677003

[B6] Butts D. A. Goldman M. S. (2006). Tuning curves, neuronal variability, and sensory coding. PLoS Biol. 4:e92. 10.1371/journal.pbio.0040092 16529529 PMC1403159

[B7] Ceylan G. Herzog M. H. Pascucci D. (2021). Serial dependence does not originate from low-level visual processing. Cognition 212, 104707–104709. 10.1016/j.cognition.2021.104709 33838523

[B8] Chen A. Gu Y. Takahashi K. Angelaki D. E. DeAngelis G. C. (2008). Clustering of self-motion selectivity and visual response properties in macaque area MSTd. J. Neurophysiol. 100, 2669–2683. 10.1152/jn.90705.2008 18753323 PMC2652227

[B9] Cicchini G. M. Mikellidou K. Burr D. C. (2018). The functional role of serial dependence. Proc. Royal Soc. B 285:20181722. 10.1098/rspb.2018.1722 30381379 PMC6235035

[B10] De Valois K. K. (1977). Spatial frequency adaptation can enhance contrast sensitivity. Vis. Res. 17, 1057–1065. 10.1016/0042-6989(77)90010-4 595415

[B11] DeYoe E. A. Van Essen D. C. (1988). Concurrent processing streams in monkey visual cortex. Trends Neurosci. 11, 219–226. 10.1016/0166-2236(88)90130-0 2471327

[B12] Dubin M. J. Duffy C. J. (2007). Behavioral influences on cortical neuronal responses to optic flow. Cerebral Cortex 17, 1722–1732. 10.1093/cercor/bhl083 17008413

[B13] Dubin M. J. Duffy C. J. (2009). Neuronal encoding of the distance traversed by covert shifts of spatial attention. Neuroreport, 20, 49–55. 10.1097/WNR.0b013e32831b44b2 19086143 PMC2691571

[B14] Fischer J. Whitney D. (2014). Serial dependence in visual perception. Nat. Neurosci. 17, 738–743. 10.1038/nn.3689 24686785 PMC4012025

[B15] Fritsche M. de Lange F. P. (2019). The role of feature-based attention in visual serial dependence. J. Vis. 19, 1–13. 10.1167/19.13.21 31770772

[B16] Fritsche M. Majorityert P. de Lange F. P. (2017). Opposite effects of recent history on perception and decision. Curr. Biol. 27, 590–595. 10.1016/j.cub.2017.01.006 28162897

[B17] Goodale M. A. Milner A. D. (1992). Separate visual pathways for perception and action. Trends Neurosci. 15, 20–25. 10.1016/0166-2236(92)90344-8 1374953

[B18] Kiyonaga A. Scimeca J. M. Bliss D. P. Whitney D. (2017). Serial dependence across perception, attention, and memory. Trends Cogn. Sci. 21, 493–497. 10.1016/j.tics.2017.04.011 28549826 PMC5516910

[B19] Kuai S. G. Chen J. Xu Z. X. Li J. M. Field D. T. Li L.. (2020). Integration of motion and form cues for the perception of self-motion in the human brain. J. Neurosci. 40, 1120–1132. 10.1523/JNEUROSCI.3225-18.2019 31826945 PMC6988997

[B20] Ma W. J. Beck J. M. Pouget A. (2008). Spiking netstudys for Bayesian inference and choice. Curr. Opin. Neurobiol. 18, 217–222. 10.1016/j.conb.2008.07.004 18678253

[B21] Manassi M. Murai Y. Whitney D. (2023). Serial dependence in visual perception: a meta-analysis and review. J. Vis. 18, 1–29. 10.1167/jov.23.8.18 37642639 PMC10476445

[B22] Matsuyoshi D. Hirose N. Mima T. Fukuyama H. Osaka N. (2007). Repetitive transcranial magnetic stimulation of human MT+ reduces apparent motion perception. Neurosci. Lett. 429, 131–135. 10.1016/j.neulet.2007.10.002 17997041

[B23] Milner D. Goodale M. A. (2006). The Visual Brain in Action (2nd Edn.). New York: Oxford University Press.

[B24] Mishkin M. Ungerleider L. G. Macko K. A. (1983). Object vision and spatial vision: two cortical pathways. Trends Neurosci. 6, 414–417. 10.1016/0166-2236(83)90190-X

[B25] Niehorster D. C. Cheng J. C. Li L. (2010). Optimal combination of form and motion cues in human heading perception. J. Vis. 10, 1–15. 10.1167/10.11.20 20884515

[B26] Or C. C. Khuu S. K. Hayes A. (2010). Moving glass patterns: asymmetric interaction between motion and form. Perception 39, 447–463. 10.1068/p5917 20514995

[B27] Pascucci D. Mancuso G. Santandrea E. Della Libera C. Plomp G. Chelazzi L.. (2019). Laws of concatenated perception: vision goes for novelty, decisions for perseverance. PLoS Biol. 17:e3000144. 10.1371/journal.pbio.3000144 30835720 PMC6400421

[B28] Pascucci D. Tanrikulu Ö. D. Ozkirli A. Houborg C. Ceylan G. Zerr P.. (2023). Serial dependence in visual perception: a review. J. Vis. 23, 1–23. 10.1167/jov.23.1.9 36648418 PMC9871508

[B29] Pavan A. Bimson L. M. Gall M. G. Ghin F. Mather G. (2017a). The interaction between orientation and motion signals in moving oriented Glass patterns. Vis. Neurosci. 34, 1–9. 10.1017/S0952523817000086 28965515

[B30] Pavan A. Ghin F. Donato R. Campana G. Mather G. (2017b). The neural basis of form and form-motion integration from static and dynamic translational Glass patterns: a rTMS investigation. NeuroImage 157, 555–560. 10.1016/j.neuroimage.2017.06.036 28633972

[B31] Souza A. S. Oberauer K. (2017). The contributions of visual and central attention to visual studying memory. Atten. Percep. Psychophys. 79, 1897–1916. 10.3758/s13414-017-1357-y 28600676

[B32] Stocker A. A. Simoncelli E. P. (2006). Noise characteristics and prior expectations in human visual speed perception. Nat. Neurosci. 9, 578–585. 10.1038/nn1669 16547513

[B33] Sun Q. Yan R. Wang J. Li L. (2022). Heading perception from optic flow is affected by heading distribution. i-Perception 13, 1–17. 10.1177/20416695221133406 36457854 PMC9706071

[B34] Sun Q. Zhan L. Z. Zhang B. Y. Jia S. W. Gong X. M. (2023). Heading perception from optic flow occurs at perceptual representation and studying memory stages with EEG evidence. Vis. Res. 208:108235. 10.1016/j.visres.2023.108235 37094419

[B35] Sun Q. Zhang H. Alais D. Li L. (2020). Serial dependence and center bias in heading perception from optic flow. J. Vis. 20, 1–15. 10.1167/jov.20.10.1 33001176 PMC7545086

[B36] Tang M. F. Dickinson J. E. Visser T. A. Badcock D. R. (2015). The broad orientation dependence of the motion streak aftereffect reveals interactions between form and motion neurons. J. Vis. 15, 4–18. 10.1167/15.13.4 26381835

[B37] Ungerleider L. G. Mishkin M. (1982). “Two cortical visual systems” in Analysis of Visual Behavior, eds. Goodale M. Ingle D. J. Mansfield R. J. W.. Cambridge, MA: MIT Press.

[B38] van Bergen R. S. Jehee J. F. (2019). Probabilistic representation in human visual cortex reflects uncertainty in serial decisions. J. Neurosci. 39, 8164–8176. 10.1523/JNEUROSCI.3212-18.2019 31481435 PMC6786811

[B39] Wang S. Y. Gong X. M. Zhan L. Z. You F. H. Sun Q. (2024). Attention influences the effects of the previous form orientation on the current motion direction estimation. Sci. Rep. 14:1394. 10.1038/s41598-024-52069-5 38228771 PMC10791700

[B40] Wang X. Y. Gong X. M. Sun Q. Li X. Y. (2022). Attractive effects of previous form information on heading estimation from optic flow occur at perceptual stage. J. Vis. 22, 1–15. 10.1167/jov.22.12.18 36413358 PMC9707032

[B41] Warren W. H. Morris M. W. Kalish M. (1988). Perception of translational heading from optical flow. J. Exp. Psychol. Human Percep. Perform. 14, 646–660. 10.1037//0096-1523.14.4.646 2974874

[B42] Warren W. H. Saunders J. A. (1995). Perceiving heading in the presence of moving objects. Perception 24, 315–331. 10.1068/p240315 7617432

[B43] Xu L. H. Sun Q. Zhang B. Li X. (2022). Attractive serial dependence in heading perception from optic flow occurs at the perceptual and postperceptual stages. J. Vis. 22, 11–25. 10.1167/jov.22.12.11 36350629 PMC9652722

[B44] You F. H. Gong X. M. Sun Q. (2023). Serial dependencies between form orientation and motion direction are asymmetric. Front. Psychol. 14, 1–11. 10.3389/fpsyg.2023.1248307 37744576 PMC10512465

